# Health-Related Quality of Life in Adults With Classical Infratentorial Superficial Siderosis: A Cross-sectional Study

**DOI:** 10.1212/WNL.0000000000201115

**Published:** 2022-11-08

**Authors:** Natallia Kharytaniuk, Amir Ala Mazaheri, Menelaos Pavlou, David J. Werring, Doris-Eva Bamiou

**Affiliations:** From the Ear Institute (N.K., D.-E.B.), Stroke Research Centre (D.W.), Department of Brain Repair and Rehabilitation, Queen Square Institute of Neurology (A.A.M.), and Department of Statistical Science (M.P.), University College London; and National Institute for Health and Care Research (N.K., D.-E.B.), University College London Hospitals Biomedical Research Centre (Deafness and Hearing Problems Theme), London; Southampton School of Medicine (A.A.M.), University of Southampton, UK.

## Abstract

**Background and Objectives:**

Infratentorial superficial siderosis (iSS) is a rare but disabling neurologic condition characterized by progressive hearing loss and balance and mobility problems. The functional decline in these neurologic domains with iSS progression is likely to adversely affect health-related quality of life (HRQoL). We studied the HRQoL of adults with iSS using 2 common generic HRQoL measures (Health Utilities Index Mark III [HUI3] and EuroQoL EQ5D [5 Level]) to determine the most affected domains and evaluate the association between HRQoL scores and disease duration.

**Methods:**

This observational study was an anonymous online survey. Following institutional Research Ethics Committee approval, we contacted dedicated international organizations, charities, and patient groups identified through online searches, social media, and collaborative networks, to distribute the study information and study link, inviting their members diagnosed with iSS to participate. Participation required access to a digital device connected to the Internet, confirmation of eligibility (aged 18 years and older and previously diagnosed with iSS), and informed consent to participate in the survey, which included study-specific questions (demographics, iSS, and hearing) and HRQoL questionnaires. Survey responses were captured by the Research Electronic Data Capture survey software and analyzed using the SPSS statistical package. Linear regression analysis was performed to investigate the association between HRQoL scores and disease duration.

**Results:**

Of 50 participants, 60% were male; the median (interquartile range [IQR]) age was 60 (15) years. The median (IQR) multiattribute scores for HUI3 and EQ5D were 0.36 (0.53) and 0.64 (0.33), respectively. The most frequently affected domains (moderate or worse category) were hearing (64%) and pain (48%) for HUI3 and mobility (54%) and pain (50%) for EQ5D. There was a weak association between disease duration and multiattribute scores for HUI3 (R = 0.353; adjusted R^2^ = 0.096; b = −0.008; *p* = 0.047) but not EQ5D.

**Discussion:**

Our findings demonstrate low HRQoL scores that capture low functional status in several domains typically affected in iSS, suggesting that iSS has a major adverse effect on quality of life in multiple functional domains. Measures of HRQoL in iSS should be included in clinical and research settings, including treatment trials.

Classical infratentorial superficial siderosis (iSS) of the CNS is a rare but disabling neurologic disorder that is associated almost invariably with hearing loss and often imbalance (ataxia) and myelopathy.^1^ Other features of iSS, which are probably underreported, include cognitive impairment, bladder and bowel dysfunction, and chronic pain.^1-3^

iSS is characterized by slow low-volume bleeding into the CSF–in most cases due to a dural defect caused by CNS trauma or surgery–which leads to hemoglobin breakdown and the generation of toxic iron species that are bound to ferritin forming hemosiderin deposits on the surfaces of the CNS structures including the cerebellum, brainstem, craniocervical junction, and spinal cord^2^ (Figure 1). Although the clinical course is highly variable, there is often progressive functional decline, with deterioration in hearing, balance, and mobility, and other symptoms.^1-5^ Treatment is predominantly aimed at identifying and repairing the source of bleeding, halting the effect of neurotoxic iron with an iron-chelating agent (e.g., deferiprone), or both.^2,6,7^

**Figure 1:**
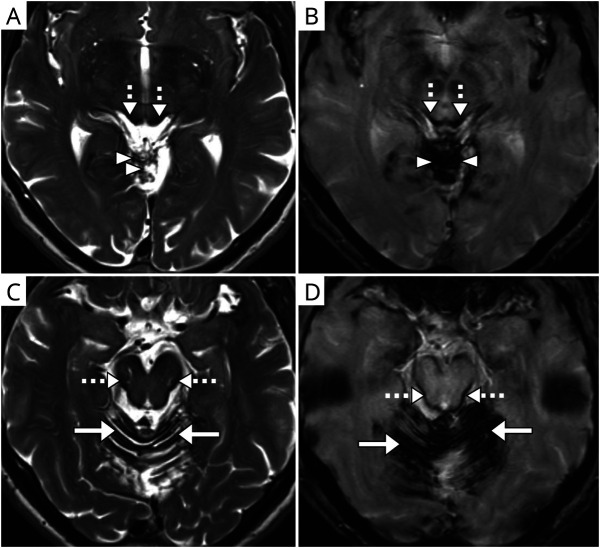
Axial Magnetic Resonance Images Showing Typical Appearances of Hemosiderin in iSS T2-weighted (A, C) and corresponding paramagnetic (B, D) sequences; images demonstrate the involvement of infratentorial regions: superior vermis (arrowheads, A, B), cerebellar folia (solid arrows, C, D), and midbrain (dashed arrows, A–D).

iSS is considered rare (defined as 1 per 2,000 EU population or affecting less than 200,000 individuals in the USA).^8-11^ The prevalence of iSS is not well described, but a few studies indicate this to be between 0.03% and 0.14%, which may be similar to that of other progressive neurodegenerative disorders such as Friedreich ataxia and some subtypes of spinocerebellar ataxia.^12-18^

The importance of evaluating the individual's perspectives of living with a rare disorder and assessing its effect on daily functioning has been previously highlighted, with measurement of quality of life suggested as a particular priority for research in rare disorders.^19-22^

The gradual functional decline in iSS, likely with disease progression, and involvement of multiple functional domains may have a marked negative impact on (health-related) quality of life (HRQoL) of individuals with iSS. Thus, an understanding of HRQoL is critical to understanding the true health effect of iSS.

Although the terms QoL and HRQoL have been used interchangeably, in this study, we used the term HRQoL defined by the UK's National Institute for Health and Care Excellence (NICE) as “a combination of a person's overall physical, mental and social wellbeing, not merely the absence of disease.”^23,24^ To measure HRQoL, they recommend using EQ5D–a generic HRQoL instrument, commonly used for benchmarking in the National Health Service.^25,26^ However, NICE also recognize the need for disease-specific or other generic instruments that capture key domains specific for the disease of interest.^27^

To the best of our knowledge, the effect of iSS on HRQoL has not been systematically studied, and no dedicated HRQoL measures for iSS exist. The aims of this study were as follows: to calculate the overall HRQoL multiattribute scores of the individuals with iSS using 2 common generic HRQoL instruments; to determine which domains demonstrate the lowest (worst affected) functional status for each instrument; and to evaluate for the association between HRQoL scores and disease duration.

## Methods

### Study Design and Setting

This observational study was an anonymous online survey that included study-specific questions and 2 validated generic HRQoL questionnaires, administered using the institution-licensed Research Electronic Data Capture (REDCap) survey platform. The online format was chosen to allow participation of individuals in a range of near and remote geographic locations and to overcome restrictions in place due to COVID-19.^28,29^

### Standard Protocol Approvals, Registrations, and Patient Consents

The study received approval by the University College London Research Ethics Committee (registration number: UCL REC 17413/001). Permissions for the academic use of the questionnaires were obtained from the EuroQOL Group for EQ5D (5-Level, English language [UK], self-administered REDCap [online] version, Registration ID 33674) and from HUInc, (Dundas, Ontario, Canada) for HUI3 (English language, self-administered, 1-week recall version).

Following the Research Ethics Committee approval, we contacted dedicated organizations, charities, and patient groups within and outside the UK, identified through online search, social media, and collaborative networks, to disseminate information about the survey and to invite their members who had been diagnosed with iSS to participate in the study. The information included a brief description of the study, a recruitment poster and participant information sheet, and the link to the study with the study-specific code.

Individuals interested in the study needed to have access to a digital device connected to the Internet to obtain information about the study and to access the study webpages. The first page contained a brief description of the study, the same participant information sheet distributed to the dedicated organizations and patient groups, and the description of the study inclusion criteria. Before commencing the survey, potential participants were asked to confirm that they had read the study information sheet and were eligible for the study: that they were aged 18 years or older and had been diagnosed with superficial siderosis by a medical professional. The term “superficial siderosis” was defined as “superficial siderosis (infratentorial) of the CNS” and described in lay language as “a very rare neurologic condition characterized by a trickle of blood through a defect in the sheath that covers the brain or spinal cord and deposition of iron on the surface of the brain, brain-related structures, and/or spinal cord, in which hearing is most often involved.”

Potential participants were provided with the online consent form and were required to agree to all sections of the consent form by ticking all the boxes. Only then were they able to proceed to the survey webpages. The survey was open and active between April 2020 and July 2021.

### Survey Contents and Participation Process

After providing the consent, the participants were taken to the study pages that included study-specific questions and the questionnaires and were able to complete the survey at their own pace including pausing the survey if necessary.

#### Study-Specific Questions

The survey-specific questions included participants' demographics (age, gender, and country of residence), iSS-specific questions (confirmation of diagnosis of iSS, age at diagnosis, whether causative event known, age at causative event, treatment, and year when commenced), and hearing-specific questions (if hearing difficulties in background noise and overall, and tinnitus present, age at onset of hearing problems, and otological history).

#### HRQoL Questionnaires

Two generic HRQoL questionnaires were included in the study. First, the Health Utilities Index Mark III (HUI3) was chosen because it includes several domains that can be affected in individuals with iSS and has been identified as a better instrument to reflect HRQoL in persons with hearing complains.^1,30,31^ Second, the EuroQOL-5D (EQ5D) 5-level was chosen because it has been widely used in the UK in a variety of settings, although it includes fewer domains with limited coverage of those likely to be affected in iSS.^32^ Both instruments provide multiattribute (utility) scores, representing overall health states, and are known to correlate with each other (Pearson *r* = 0.7) including in a cohort with hearing impairment.^31,33^

The HUI3 and EQ5D questionnaires were presented to the participants in a fixed order. The HUI3 questionnaire includes 8 domains (attributes) and consists of 15 questions of 5–6 levels, which are converted into disability categories and utility scores.^30,34^ The HUI3 domains and levels include (from Horsman et al.^34^):

1. Vision: from “able to see well enough to read ordinary newsprint and recognize a friend on the other side of the street, without glasses or contact lenses” (level 1) to “unable to see at all” (level 6);
2. Hearing: from “able to hear what is said in a group conversation with at least 3 other people, without a hearing aid” (level 1) to “unable to hear at all” (level 6);
3. Speech: from “able to be understood completely when speaking with strangers or friends” (level 1) to “unable to be understood when speaking to other people (or unable to speak at all)” (level 5);
4. Ambulation: from “able to walk around the neighbourhood without difficulty, and without walking equipment” (level 1) to “cannot walk at all” (level 6);
5. Dexterity: from “full use of 2 hands and 10 fingers” (level 1) to “limitations in use of hands or fingers, requires the help of another person for all tasks (not independent even with use of special tools)” (level 6);
6. Emotion: from “happy and interested in life” (level 1) to “so unhappy that life is not worthwhile” (level 5);
7. Cognition: from “able to remember most things, think clearly and solve day to day problems” (level 1) to “unable to remember anything at all, and unable to think or solve day to day problems” (level 6);
8. Pain: from “free of pain and discomfort” (level 1) to “severe pain that prevents most activities” (level 5);

The HUI3 outputs included the following: (1) single-attribute disability categories and utility scores and (2) multiattribute disability categories and utility scores. The scores' range of values was from 0.00 to 1.00 for single attributes (from the worst to perfect functional state), whereas the range of values for multiattribute scores, representing the overall HRQoL states, was from −0.36 to 1, with 0 equal to “being dead,” 1 being “perfectly healthy,” and less than zero scores representing “worse than dead” states.^30^ Score differences of ≥0.03 were considered clinically significant.^34^

EQ5D (5-level) assesses HRQoL in 5 domains (dimensions) and in 5 categories (levels), which include (from Herdman et al.^35^) the following:

1. Mobility: from “I have no problems in walking about” (level 1) to “I am unable to walk about” (level 5);
2. Self-care: from “I have no problems washing or dressing myself” (level 1) to “I am unable to wash or dress myself” (level 5);
3. Usual activities: from “I have no problems doing my usual activities” (level 1) to “I am unable to do my usual activities” (level 5);
4. Pain/discomfort: from “I have no pain or discomfort” (level 1) to “I have extreme pain or discomfort” (level 5);
5. Anxiety/depression: from “I am not anxious or depressed” (level 1) to “I am extremely anxious or depressed” (level 5);

Each domain is represented by a number corresponding to the category of the perceived problem, and a unique health state can be derived from combinations of the categories and the domains. Other EQ5D outcome measures include respondents' self-perceived health states represented using visual analog scale (VAS) provided by each participant as a number ranging from 0 to 100 (worst to best imaginable health state), and multiattribute (utility) scores derived using a country-specific value set reflecting the country-specific differences in values attributed to the health states.^25^ The UK tariff (crosswalk model from EQ5D 3-level) was used to calculate the utility scores for all participants irrespective of their country of residence.^36-38^ This was performed because a larger proportion of participants indicated the UK as their country of residence (followed by the USA). Participants' responses could not be standardized for demographic and geographic differences. The range of the UK tariff values was from −0.594 to 1.^37,39^ The US tariff (value range from −0.109 to 1) was included for comparative purposes only.^40,41^ The scores of 0 and 1 equalled to “being dead” and “perfectly healthy,” respectively, and “worse than dead” states were represented by less than zero scores.

### Data Collection

The data collected included coded answers to the study-specific questions and to the questionnaires. The study data were anonymous at the outset; therefore, no personally identifiable information was collected. Incomplete and duplicate entries were excluded from the analysis and not reported.

### Data Availability

Fully anonymized data will be available from the institution's research data repository (doi.org/10.5522/04/19846924) and will be stored for the duration stipulated by the institution's research data storage policy.

### Statistical Analysis

Statistical analysis was performed using SPSS version 27 (IBM Corp., Armonk, NY). A coded SPSS file with the study data outputs was generated by REDCap following the study closure. Participants' characteristics were summarized using descriptive statistics. Frequencies and percentages were reported for categorical data. All measures were assessed for normality using the Shapiro-Wilk test (*p* values >0.05 confirming assumptions of normal distribution). The mean scores with SD were reported for continuous data with normal distribution; the median scores with interquartile ranges (IQRs) were reported for continuous data that seemed to deviate from normality.

Nonparametric tests were used for the analysis of data that seemed to violate assumptions of normal distribution. The Mann-Whitney *U* test was performed to assess the difference in the mean ranks of the utility scores based on gender, treatment status, the presence of hearing problems and tinnitus and between the multiattribute scores of EQ5D and HUI3. The significance level was set at 0.05.

Linear regression analysis was performed to study the associations between the instruments' scores and disease duration. Although the outcomes (EQ5D scores and calculated disease duration) exhibited slight skewness, the residuals from the model fits were assessed for normal distribution using histograms and probability (P-P) plots. There were no missing data within the HRQoL instruments.

## Results

### Demographics and Study-Specific Questions

Of 50 participants included in the study, 60% were male; the median age was 60 (IQR 15) years. Most participants (64%) reported the UK as their country of residence, followed by the USA (32%). English was the primary language for most (98%) (Table 1).

**Table 1:**
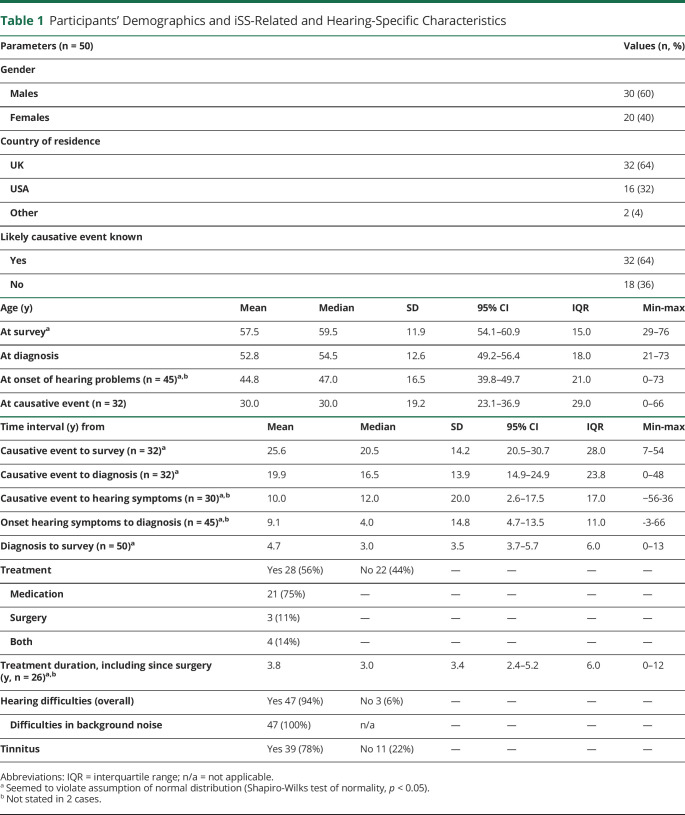
Participants' Demographics and iSS-Related and Hearing-Specific Characteristics

The likely causative event for developing iSS was known in 64% of cases; the mean age during the presumed causal event was 30 (SD 19), with 2 participants being younger than 1 year during the presumed event (Table 1).

The mean age during iSS diagnosis was 53 (SD 13) years. The median duration between the likely causative event and the diagnosis was 17 (IQR 24) years. In only 6% of cases, the diagnosis was achieved within the same year of the likely causative event.

Most (94%) participants reported hearing difficulties, including in the presence of background noise (Table 1). The median age at the onset of hearing problems was 47 (IQR 21) years. A family history of early-onset hearing loss (not related to age) was reported by 14% participants. The median time interval between the onset of hearing symptoms and the diagnosis was 4 (IQR 11) years; 12% participants had the onset of hearing problems after the diagnosis (all within 3 years), and 10% participants reported the onset of hearing problems within the same year as diagnosis. The median time interval between the likely causative event and the onset of hearing symptoms was 12 (IQR 17) years, but some hearing symptoms preceded the likely causative event in 12% of cases.

More than half (56%) of participants reported they had received treatment for iSS, 75% of whom were receiving medical treatment; 14% participants had received both surgery and medication. The mean treatment duration at the study participation was 4 (SD 3) years.

### HRQoL Measures

#### HUI3

**Figure 2:**
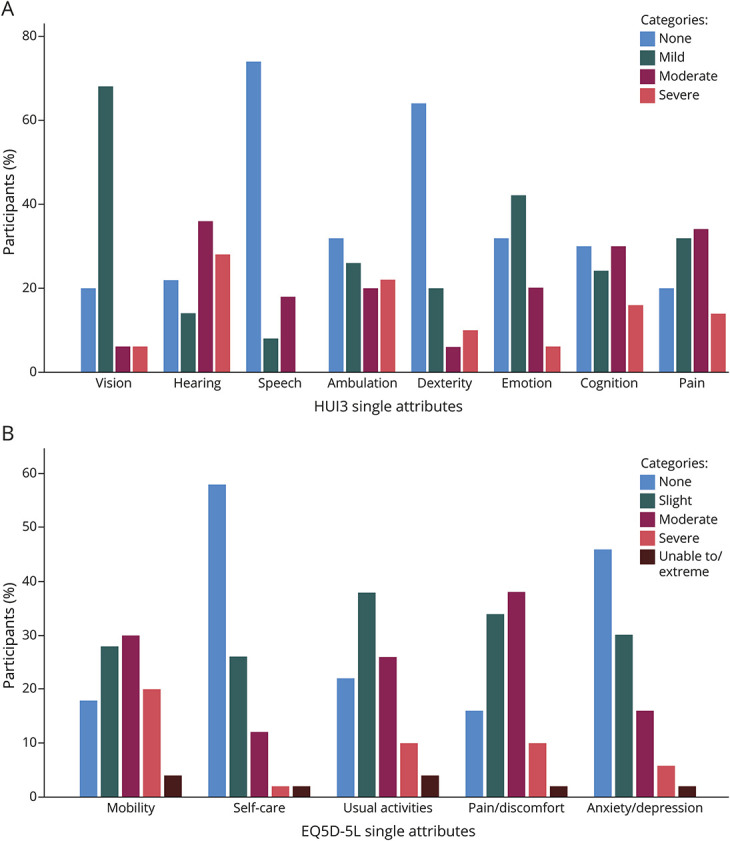
Single Attributes by Categories for Both Instruments A) Health Utilities Index Mark III (HUI3) and (B) EuroQOL-5D 5-level (EQ5D-5L).

The most frequently affected domains (moderate or worse category) were hearing (64%), pain (48%), cognition (46%), and ambulation (42%) (Figure 2, Table 2). These domains also demonstrated the lowest single-attribute scores (median, IQR): hearing (0.71, 0.54) and ambulation (0.83, 0.33), followed by cognition (0.92, 0.30) and pain (0.92, 0.15). Less frequently involved domains were emotion (26%), speech (18%), dexterity (16%), and vision (12%).

**Table 2:**
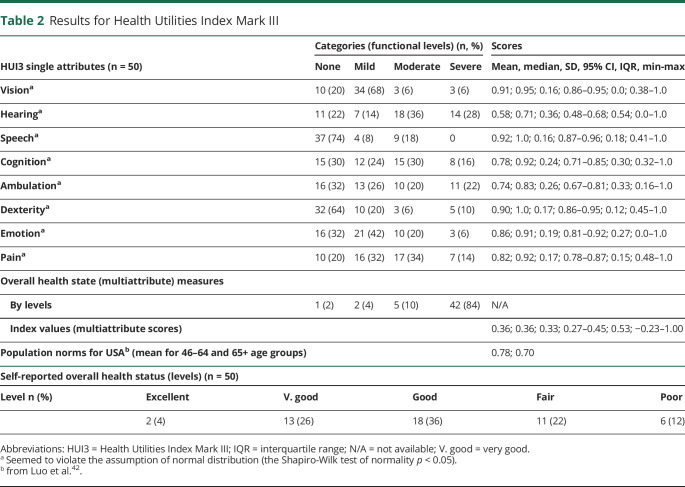
Results for Health Utilities Index Mark III

The mean HUI3 multiattribute scores were 0.36 (SD 0.33); scores less than zero were observed in 16% of cases; the highest score of 1 was reached in 2% of cases. The mean rank comparison (the Mann-Whitney *U* test) of HUI3 multiattribute scores identified statistically significant lower scores for female individuals (n = 20; U = 196.0; z = −2.06; *p* = 0.039) and for individuals with hearing problems (n = 47; U = 138.0; z = 2.76; *p* = 0.006), but not based on treatment status (n = 28; U = 308.0; z = 0.0; *p* = 1.000) or the presence of tinnitus (n = 39; U = 141.0; z = −1.72; *p* = 0.085).

#### EQ5D

Analysis of the EQ5D categories (ranging from no problems to extreme problems) demonstrated that the most affected domains (moderate or worse category) were mobility (54%), pain (50%), and usual activities (40%); the least affected domain was self-care (16%) (Figure 2, Table 3).

**Table 3:**
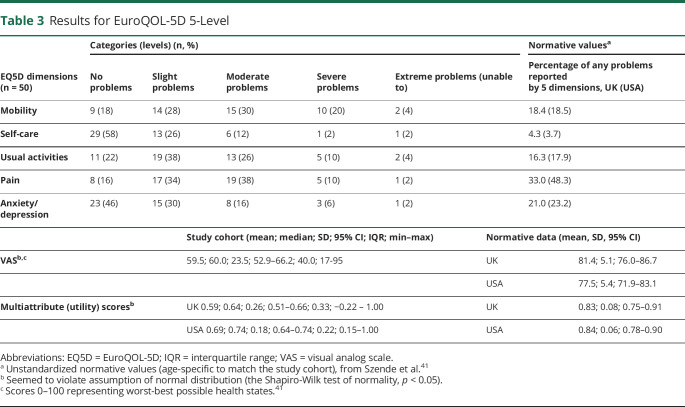
Results for EuroQOL-5D 5-Level

The median values for the EQ5D multiattribute scores were 0.64 (IQR 0.33) and for the VAS were 60 (IQR 40); scores less than zero were observed in 4% participants; the highest score of 1 was observed in 6% participants.

The mean rank comparison (the Mann-Whitney *U* test) of EQ5D multiattribute scores identified statistically significant lower scores for individuals with hearing problems (n = 47; U = 122.0; z = 2.10; *p* = 0.035) and those with tinnitus (n = 39; U = 76.5; z = −3.23; *p* = 0.001), but not based on gender (n = 20; U = 201.5; z = −1.95; *p* = 0.051) or treatment status (n = 28; U = 352.0; z = 0.86; *p* = 0.39).

### Analysis of HRQoL Scores and Disease Duration

The multiattribute scores for HUI3 (mean rank = 39.9) seemed to be lower than the EQ5D multiattribute scores (mean rank = 61.1); the difference was statistically significant: U = 1778.5, z = 3.64; *p* < 0.001.

Linear regression analysis identified a strong association between both instruments' multiattribute scores despite the statistically significant difference in their mean ranks: R = 0.635; adjusted R^2^ = 0.391; b = 0.503; 95% CI 0.325–0.680; and between the HUI3 multiattribute scores and EQ5D VAS values: R = 0.665; adjusted R^2^ = 0.430; b = 0.009; 95% CI 0.006–0.012 (Table 4).

**Table 4:**
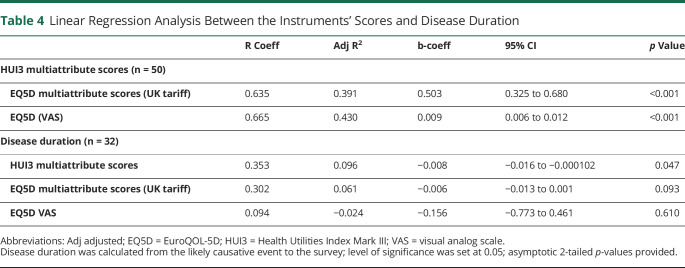
Linear Regression Analysis Between the Instruments' Scores and Disease Duration

There was a weak association between disease duration and multiattribute scores for HUI3 (R = 0.353; adjusted R^2^ = 0.096; b = −0.008; 95% CI −0.016 to −0.000102) but not EQ5D (Table 4). Although the EQ5D scores and disease duration demonstrated slightly skewed data distribution, the residuals from the linear regression model fits demonstrated approximately normal distribution (assessed using histograms and probability [P-P] plots).

## Discussion

**Figure 3:**
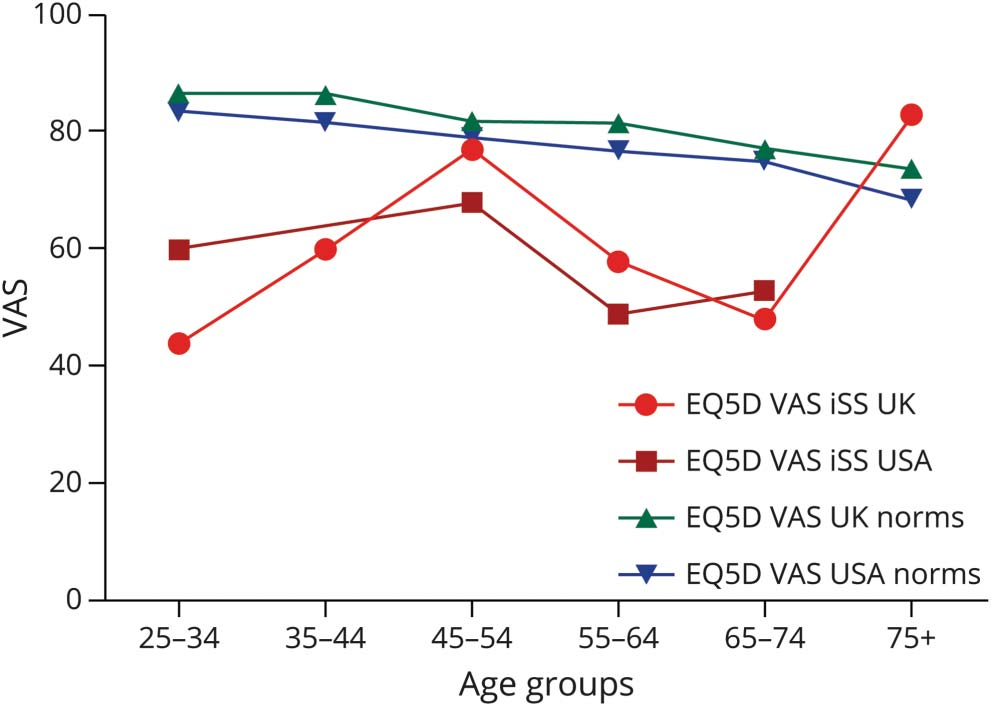
EuroQOL-5d (EQ5D) Visual Analogue Scale (VAS) Values for the UK and the USA Cohorts Respective country norms provided from Janssen et al.^38^

To the best of our knowledge, this is the first study to quantitatively and systematically capture HRQoL in iSS. Using 2 common validated generic measures (HUI3 and EQ5D), we found that HRQoL scores were lower than population norms for both instruments (Figure 3, Tables 2 and 3).^41-43^ HUI3 identified moderate or worse overall functional status in 94% of participants. Hearing difficulty is the most frequently reported feature of iSS, which was observed in our study, with the worst functional levels and utility scores observed in the HUI3 hearing domain.

The study findings also demonstrated the effect of iSS on multiple functional domains including hearing, cognition, ambulation, and pain, all of which have been reported as being affected in patients with iSS.^1-3^ These findings suggest that pain and cognitive impairment in iSS may have a similar prevalence as problems with mobility and hearing, so may be key aspects of the iSS clinical syndrome. Indeed, cognitive impairment has been observed in 50% of patients with iSS studied in the clinical setting.^3^

EQ5D analysis identified mobility and pain as the most frequently affected domains, in keeping with the HUI3 affected domains of ambulation and pain. However, the EQ5D may underestimate the effect of iSS on HRQoL for individuals in whom hearing or cognitive problems predominate because these domains are not included in the EQ5D. Thus, in the absence of iSS-specific measures, HUI3 might be a more suitable instrument for assessing HRQoL in adults with iSS, in line with the NICE recommendations for tailored HRQoL instruments.^27^

Furthermore, the HUI3's potentially superior sensitivity to measure HRQoL in iSS is probably also reflected in the instruments' multiattribute scores that were significantly lower than for EQ5D. More HUI3 multiattribute scores were observed to be less than zero than for EQ5D; however, this might be due to the previously described floor and ceiling effects.^33,44^

The multiattribute scores seemed to be worse for participants with hearing problems (for both EQ5D and HUI3) and with tinnitus (for EQ5D). It possible that despite not including hearing as a domain, the EQ5D anxiety/depression domain was partially able to capture the effect of tinnitus on HRQoL because it is well known to be related to anxiety and stress.

The scores observed in our cohort seemed to be similar to those derived from patients with several other complex neurologic conditions, such as patients with moderate disability from traumatic brain injury (mean scores of 0.51 [EQ5D, Dutch tariff] and HUI3 0.48 for moderate and 0.33 lower moderate disability), although direct comparisons between the studies could not be made due to demographic differences (age, country of study, and tariffs used) and the severity of disorders for which HUI3 and EQ5D were reported.^45^ The mean EQ5D multiattribute scores (and VAS values) in our study also seemed to be similar to those reported for motor neuron disease: 0.57 (60) (albeit in an older cohort),^46^ and in stroke patients of similar age: 0.52 (53).^37^

The strength of this study is that it is first of its kind to measure HRQoL in iSS. The anonymous online format facilitated reaching out to wider patient population groups and optimizing recruitment opportunities. This approach may also have reduced bias associated with face-to-face completion of questionnaires.^47^ The sample size in our study should be considered as large in view of iSS rarity because most of studies' cohorts with iSS are in single or teen figures.

There are several limitations to our study. We were unable to independently verify the clinical and radiologic diagnosis of iSS, including brain and spine MRI. To mitigate this, participants were asked twice to confirm their diagnosis: first, in the consent form and then, again within the study-specific questions. Participants needed to have access to a digital device and Internet, which might have introduced selection bias, whereas order bias may have resulted from presentation of questionnaires in a fixed order and recall bias due to the retrospective nature of study specific questions. The study was conducted predominantly focusing on problems with hearing because it is often the most common and earliest symptom reported by individuals with iSS; we did not collect data on urinary and bowel problems, and they are likely not to have been captured by the 2 instruments used in the study.

Although our results demonstrated the presence of association between the HUI3 multiattribute scores and disease duration, this analysis should be interpreted with caution because the self-perceived HRQoL may be influenced by other factors, which might not have been accounted for in this study—including the individual's coping strategies and abilities.

Owing to the study design, the collected outcomes could not be compared with objective clinical measures of impairments reflecting the severity of iSS clinical syndrome. Further longitudinal studies are needed to correlate HRQoL utility scores with clinical measures and determine whether HUI3 can capture small but clinically significant changes over time.

This study found markedly reduced HRQoL scores in iSS, which may be comparable with other complex neurologic conditions, highlighting an unmet need for health care resources to tackle the consequences of iSS. Our study identified potential advantages of HUI3 over EQ5D in iSS including assessment of hearing, lower multiattribute scores, and correlation of HUI3 scores with disease duration (suggesting face validity). Because iSS-specific HRQoL measures are lacking, we therefore propose the use of HUI3 (or a combination of HUI3 and EQ5D) to capture the effect of iSS on HRQoL.

## Glossary

EQ5D: EuroQOL-5D
HRQoL: health-related quality of life
HUI3: Health Utilities Index Mark III
IQRs: interquartile ranges
iSS: infratentorial superficial siderosis
NICE: National Institute for Health and Care Excellence
REDCap: Research Electronic Data Capture
VAS: visual analog scale
